# Topical Anti-Inflammatory Effects of Isorhamnetin Glycosides Isolated from *Opuntia ficus-indica*

**DOI:** 10.1155/2015/847320

**Published:** 2015-03-02

**Authors:** Marilena Antunes-Ricardo, Janet A. Gutiérrez-Uribe, Carlos Martínez-Vitela, Sergio O. Serna-Saldívar

**Affiliations:** Centro de Biotecnología-FEMSA, Tecnológico de Monterrey, Avenida Eugenio Garza Sada 2501 Sur, 64849 Monterrey, NL, Mexico

## Abstract

*Opuntia ficus-indica* (OFI) has been widely used in Mexico as a food and for the treatment of different health disorders such as inflammation and skin aging. Its biological properties have been attributed to different phytochemicals such as the isorhamnetin glycosides which are the most abundant flavonoids. Moreover, these compounds are considered a chemotaxonomic characteristic of OFI species. The aim of this study was to evaluate the effect of OFI extract and its isorhamnetin glycosides on different inflammatory markers *in vitro* and *in vivo*. OFI extract was obtained by alkaline hydrolysis of OFI cladodes powder and pure compounds were obtained by preparative chromatography. Nitric oxide (NO), cyclooxygenase-2 (COX-2), tumor necrosis factor- (TNF-) *α*, and interleukin- (IL-) 6 production were measured. NO production was tested in lipopolysaccharide-stimulated RAW 264.7 cells while *in vivo* studies were carried on croton oil-induced ear edema model. OFI extract and diglycoside isorhamnetin-glucosyl-rhamnoside (IGR) at 125 ng/mL suppressed the NO production *in vitro* (73.5 ± 4.8% and 68.7 ± 5.0%, resp.) without affecting cell viability. Likewise, IGR inhibited the ear edema (77.4 ± 5.7%) equating the indomethacin effects (69.5 ± 5.3%). Both IGR and OFI extract significantly inhibited the COX-2, TNF-*α*, and IL-6 production. IGR seems to be a suitable natural compound for development of new anti-inflammatory ingredient.

## 1. Introduction

The skin is the first line of defense against the external environment and xenobiotic agents. It interacts with both endogenous and environmental oxidant species which are involved in the pathogenesis of many inflammatory skin diseases [1–4]. Cutaneous injury causes keratinocyte activation, proliferation, and releasing of inflammatory mediators such as nitric oxide (NO), tumor necrosis factor alpha (TNF-*α*), and interleukin- (IL-) 1 and IL-6 [5, 6].

Different inflammation models have been used to understand the anti-inflammatory mechanisms of synthetic drugs or compounds from natural sources [7]. Lee et al. [8] and Yoon et al. [9] observed that the extracts of* Chrysanthemum indicum* and* Scutellaria baicalensis* induced anti-inflammatory responses* in vivo* and* in vitro*. These effects were evidenced by a reduction on the NO production, cytokines, chemokine, and growth factors, along with different histopathological parameters. Additionally, topical formulations which combine naturally occurring compounds and nutritional supplements have been developed and these have shown great potential to inhibit oxidative stress and skin inflammation [10].

The ear edema inflammation model has been widely used to evaluate the anti-inflammatory potential of natural compounds. The topical application of croton oil as an irritant agent increases vascular permeability and induces the synthesis of arachidonic acid metabolites and the expression of COX-2, IL-1*β*, TNF-*α*, and the adhesion molecule ICAM-1 [11].

Different flavonoids-rich extracts and isolated flavonoids have been tested* in vitro* and* in vivo* to evaluate their anti-inflammatory effects. For example, a flavonoid-enriched fraction obtained from* Cayaponia tayuya* roots (0.5 mg/ear) showed an inhibition of 66% in acute TPA-induced edema in mouse ears. When the extract was tested at 22.30 *μ*g/ml on RAW 264.7 macrophages, it inhibited the expressions of iNOS and COX-2 by 97% and 65%, respectively [15]. Likewise, flavonoids contained in a flavonoid-enriched fraction (50 *μ*g/mL) extracted from the rhizomes of* Sophora flavescens *also showed* in vitro *inhibitory effects greater than 50% on the NO production [16]. Isorhamnetin, kaempferol, and quercetin inhibited* i*NOS protein and* m*RNA expression when tested in macrophages stimulated with lipopolysaccharide (LPS) [17]. Also, these compounds inhibited the activation of nuclear transcription factor-*κ*B (NF-*κ*B).

In nature, flavonoids are generally attached to sugar residues that modify the mechanism of absorption and their ability to enter cells or to interact with transporters and cellular lipoproteins [18]. Therefore there are differences among the biological effects exerted by flavonoid glycosides according to the type, number, and position of sugar moieties [19, 20]. de Melo et al. [21] showed that kaempferol 3-*O*-*β*-glucopyranoside-7-*O*-*α*-rhamnopyranoside and kaempferol 7-*O*-*α*-rhamnopyranoside inhibited the croton oil-induced ear edema by 46.5% and 33.3%, respectively. Likewise, kaempferol 3-*O*-*β*-D-apiofuranosyl-(1→2)-*α*-L-arabinofuranosyl-7-*O*-*α*-L-rhamnopyranoside (10 *μ*M) showed better inhibitory effects on TNF-*α* and IL-12 production than kaempferol 3-*O*-*β*-D-apiofuranosy-(1→4)-*α*-L-rhamnopyranosyl-7-*O*-*α*-L-rhamnopyranoside (40 *μ*M) [22].

*Opuntia ficus-indica *(OFI) has been used in Mexico as a food and as a remedy against different health disorders related to skin such as inflammation [23, 24]. This plant is a natural source of flavonoids, mainly isorhamnetin glycosides [12–14, 25]. Isorhamnetin and isorhamnetin-3-*O*-galactoside possess* in vitro* anti-inflammatory activity [26, 27].

Considering all the above, the aim of this research work was to evaluate the* in vitro* and* in vivo *anti-inflammatory effects of an OFI extract and compare the bioactivity of different isorhamnetin glycosides isolated from this source. OFI extract and isolated isorhamnetin diglycosides and triglycosides were tested on RAW 264.7 macrophage cells in order to evaluate their effect on NO production. Later, these compounds were tested in a rat ear edema inflammation model to evaluate their effect on COX-2, TNF-*α* and, IL-6 levels.

## 2. Materials and Methods

### 2.1. Reagents

Dulbecco's Modified Eagle Medium: Nutrient Mixture F-12 (DMEM-F12), ampicillin/streptomycin, and phosphate-buffered saline (pH 7.4) were purchased from Gibco Invitrogen (Carlsbad, CA). Trypsin-EDTA (0.25%) and fetal bovine serum were obtained from HyClone Thermo Scientific (Logan, UT). Triton X-100 was acquired from Research Organics (Cleveland, OH). Lipopolysaccharides (LPS) from* Salmonella enterica serotype typhimurium* L7261, as well as croton oil, indomethacin, isorhamnetin standard, and formic acid solution HPLC grade were purchased from Sigma-Aldrich (St. Louis, MO). Chromatography grade water and methanol (VWR International LLC, West Chester, PA) were used for high-pressure liquid chromatograph equipped with a photodiode array detector (HPLC-PDA) and liquid chromatograph/mass selective detector time-of-flight (LC/MSD TOF) analysis.

### 2.2. Obtaining* O. ficus-indica* Extract

The taxonomic identification of* Opuntia ficus-indica* was done by Ph.D. Rigoberto E. Vázquez-Alvarado at the School of Agronomy of Universidad Autónoma de Nuevo León (UANL), México. OFI cladodes were harvested at 7 months and grown in the region of Montemorelos, Nuevo Leon, México, and then they were processed into powder according to Santos-Zea et al. [14]. OFI extract was obtained by alkaline hydrolysis following the method previously reported by Antunes-Ricardo et al. [12].

### 2.3. Identification, Quantification, and Purification of Isorhamnetin Glycosides in OFI Extract

Identification and quantification of isorhamnetin glycosides were performed according to the method described by Antunes-Ricardo et al. [12] using HPLC-PDA (Agilent 1100 Series Santa Clara, CA). Purification of isorhamnetin glycosides was done by semipreparative chromatography according to the method described by Antunes-Ricardo et al. [12] using a semipreparative Zorbax SB-C18 (9.4 × 250 mm, 5 *μ*m) column at 17°C, flow rate of 2.0 mL/min, and injection volume of 50 *μ*L. Water with 0.1% formic acid (A) and methanol 80% (B) were used as mobile phases, starting with 35% of B and increasing to 60% and 90% after 2 and 19 min, respectively, and then decreasing to 0% B for the next 3 min. Percentages of purity of 90% or more were obtained for the isorhamnetin glycosides. LC/MSD TOF technology (Model G1969A Agilent 1100 Santa Clara, CA) was used to identify the isorhamnetin glycosides according to the method described by Santos-Zea et al. [14].

### 2.4. RAW 264.7 Cell Culture

RAW 264.7, a murine macrophage cell line, was obtained from the American Type Culture Collection (ATCC, Manassas, VA). Cells were cultured in petri plates with Dulbecco's Modified Eagle Medium (DMEM) supplemented with 10% fetal bovine serum (FBS) and 1% antibiotic and incubated at 37°C and 5% of CO_2_ (NuAire, Plymouth, MN). To evaluate the effects of an OFI extract and isolated isorhamnetin glycosides on nitric oxide production, cells were plated in 96-well plate (5 × 10^4^ cells/well) and allowed to adhere for 4 h. After that, different concentrations (25, 50, or 125 ng isorhamnetin equivalents (IsoEq/mL) of the compounds were added and incubated for another 24 h. Following, half of wells were stimulated with LPS at 10 *μ*g/mL (final concentration) while the other half were used as control for each sample.

### 2.5. Measurement of Nitrite Concentration

Production of nitric oxide (NO) was measured by nitrites determination, which is a stable and nonvolatile breakdown product of NO. After incubation with the different treatments, 100 *μ*L of supernatant was transferred to another 96-well plate to measure nitrites concentration using the Griess Reagent System (Promega, Madison, WI) at 550 nm. Data was expressed as percentage of inhibition of NO production. The remaining cells and medium in original plate were used to measure cell viability.

### 2.6. Measurement of RAW 264.7 Cell Viability

Cell viability was determined by CellTiter 96 AQueous One Solution Cell Proliferation Assay (Promega, Madison, WI). Absorbance was measured at 490 nm with a 96-well microplate reader (Synergy HT, Bio-Tek, Winooski, VM). Cell viability was calculated by dividing the absorbance of cells treated by the absorbance of control cells, and this ratio was expressed as percentage.

### 2.7. Animals

Male Wistar rats (250–300 g) were purchased from Bioinvert (Mexico, DF, Mexico). Animals were divided into seven groups (*n* = 8) and kept in a room with controlled temperature (25°C) and relative humidity (50 ± 5%) for 12 h light/dark cycles with food and water* ad libitum*. The Institutional Committee on Care and Use of Experimental Animals at the Tecnológico de Monterrey approved this study with the protocol number 2014-RE-001.

### 2.8. Acute Ear Edema Induced by Croton Oil

Experiments were carried out as described previously [28, 29]. Cutaneous inflammation was induced by the application of 5% croton oil (20 *μ*L) in acetone in the outer surface of the right ear in rats. The left ear received an equal volume of vehicle (acetone). After 30 minutes, OFI extract, isorhamnetin glycosides, isorhamnetin standard at dose of 1.0 *μ*moL IsoEq/ear, or indomethacin (1.0 *μ*moL/ear) was applied topically in the right ear in the test groups. The left ear of each experimental subject of the test groups received an equal volume of the vehicle. The control group only received vehicle as treatment. Test compounds and vehicle were applied by an automatic micropipette. Animals were euthanized by cervical dislocation 4 hours after croton oil application. Both ears were removed with a metal punch (6 mm diameter disc) and weighed (APX-100, Denver Instrument, Denver, CO). Ear edema was calculated by subtracting the weight of the left ear (untreated) from the right ear (treated). The results were expressed as percentage of inhibition of ear edema with respect to the control group according to the following: inhibition (%): [(ear edema of control group) − (ear edema of treated group)] × 100/[ear edema of control group].

### 2.9. Measurement of COX-2, TNF-*α*, and IL-6 in the Ear Edema Model Induced by Croton Oil

Ear biopsy samples were pooled by group and homogenized vigorously (Tissue Tearor, BioSpec Products, Racine, WI) in 1.5 mL of extraction buffer (containing 10 mM Tris pH 7.4, 150 mM NaCl, and 1% Triton X-100) per gram of sample. The homogenates were centrifuged at 13,000 g for 10 minutes at 4°C in a Microfuge 22R centrifuge (Beckman Coulter, Atlanta, GA) and the supernatants were stored at −80°C until being analyzed. The inhibition of COX-2 activity was determined by the COX Colorimetric Inhibitor Screening Assay (Cayman Chemical, Ann Arbor, MI). The levels of TNF-*α* and IL-6 in the supernatants were measured using commercially available sandwich enzyme-linked immunosorbent assay (ELISA) kits (Invitrogen Corp., Camarillo, CA) according to the manufacturer's instructions. The results were expressed as percentage of inhibition with respect to control.

### 2.10. Statistical Analysis

All measurements were performed at least in triplicate and results were expressed as mean ± standard deviation. Statistical analyses were performed with the JMP 8.0 software (SAS Institute Inc., Cary, NC). Data was analyzed by ANOVA methodology followed by Tukey's HSD tests with a significance level of *P* < 0.05.

## 3. Results

### 3.1. Identification and Quantification of Isorhamnetin Glycoside in OFI Extract

**Figure 1:**
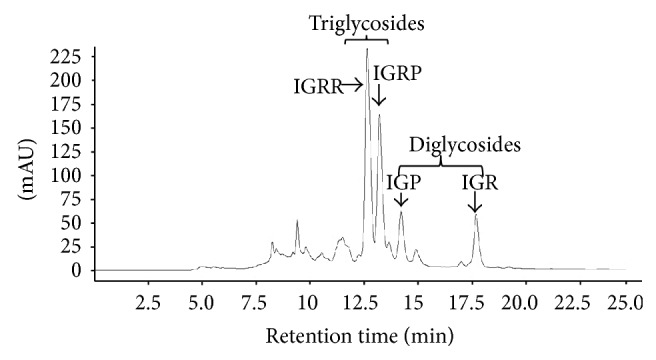
Chromatogram obtained at 365 nm showing the most abundant isorhamnetin glycosides found in* Opuntia ficus-indica* extract. IGRR: isorhamnetin-glucosyl-rhamnosyl-rhamnoside; IGRP: isorhamnetin-glucosyl-rhamnosyl-pentoside; IGP: isorhamnetin-glucosyl-pentoside; IGR: isorhamnetin-glucosyl-rhamnoside.

The most abundant isorhamnetin diglycosides were isorhamnetin-glucosyl-pentoside (IGP) and isorhamnetin-glucosyl-rhamnoside (IGR) whereas isorhamnetin-glucosyl-rhamnosyl-rhamnoside (IGRR) and isorhamnetin-glucosyl-rhamnosyl-pentoside (IGRP) were the most abundant triglycosides (Figure 1).  Table 1 shows quantification of isorhamnetin glycosides in the OFI extract and their identification by comparison with previous reports of lambda maxima (UV/VIS) obtained by HPLC and* m/z* [M+H] obtained by mass spectrometry (see Supplementary Figures S1 and S2 in Supplementary Material available online at http://dx.doi.org/10.1155/2015/847320). Along with the molecular ion, sodium adducts were obseved in each mass spectra. Ionization conditions allowed the detection of fragments generated by the loss of three, two and one sugar moieties observed in the triglycosides mass spectrum (Figure S1); likewise, fragments generated by the loss of two and one sugar moieties were observed in the diglycosides mass spectrum (Figure S2). The* m/z* [317.05] corresponding to isorhamnetin aglycone appears in all mass spectrum.

### 3.2. Effect of OFI Extract and Isorhamnetin Glycosides in Cell Viability and NO Production in RAW 264.7 Macrophage Cells Stimulated by LPS

Isorhamnetin triglycosides, IGRR, and IGRP did not affect the cell viability at any of the concentrations tested (Table 2). In contrast, IGP, IGR, and OFI extract showed a slight decrease in cell viability (84.9 ± 4.0%, 81.6 ± 3.0%, and 89.9 ± 1.9%, resp.) when tested at 125 ng IsoEq/mL.

Regarding the inhibitory effects of isorhamnetin glycosides and OFI extract on NO production (Table 3), data showed that OFI extract was more potent inhibitor than isorhamnetin at all tested concentrations, exhibiting 73.5 ± 4.8% as the maximum inhibition value at 125 ng IsoEq/mL.

At 25 ng IsoEq/mL, IGRP and diglycosides IGP and IGR were better inhibiting NO production showing 53.2 ± 4.7%, 57.4 ± 9.1%, and 60.0 ± 3.4% of inhibition, respectively. At the same concentration, IGR showed also better effect inhibiting NO production than OFI extract. But when the concentration was increased to 50 and 125 ng IsoEq/mL, IGR and OFI extract increased NO inhibition by 35.9% and 32.7%, respectively. Although isorhamnetin bioactivity significantly increased with concentration, it was still the least potent inhibitor of NO production (47.6 ± 5.8%).

### 3.3. Effect of Isorhamnetin Glycosides and OFI Extract in Acute Ear Edema Induced by Croton Oil

**Figure 2:**
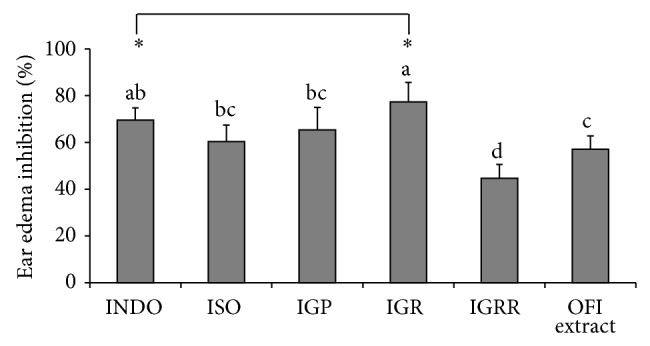
Effect of indomethacin (INDO), isorhamnetin (ISO), isorhamnetin glycosides, and* Opuntia ficus-indica* (OFI) extract on croton oil-induced rat ear edema. IGP: isorhamnetin-glucosyl-pentoside; IGR: isorhamnetin-glucosyl-rhamnoside; IGRR: isorhamnetin-glucosyl-rhamnosyl-rhamnoside. ^a, b, c, d^Different letters denote significant differences among treatments (*P* < 0.05). ^*^Treatment with highest potential to inhibit ear edema.

The diglycoside IGR and indomethacin showed the greatest potential to inhibit the ear edema induced by croton oil, 77.4 ± 5.7% and 69.5 ± 5.3%, respectively (Figure 2). Inhibition exhibited by IGP was slightly lower (65.3 ± 5.9%) than the observed for IGR but similar to the observed for isorhamnetin. The isorhamnetin triglycoside IGRR showed lower inhibitory effect on ear inflammation (44.7 ± 8.2%) than IGR and OFI extract (57.1 ± 9.7%). In this work isorhamnetin glycosides IGP and IGR, but not IGRR, showed better topical anti-inflammatory potential than OFI extract.

### 3.4. Effect of Isorhamnetin Glycosides and OFI Extract in Proinflammatory COX-2 Activity and TNF-*α* and IL-6 Production

**Figure 3:**
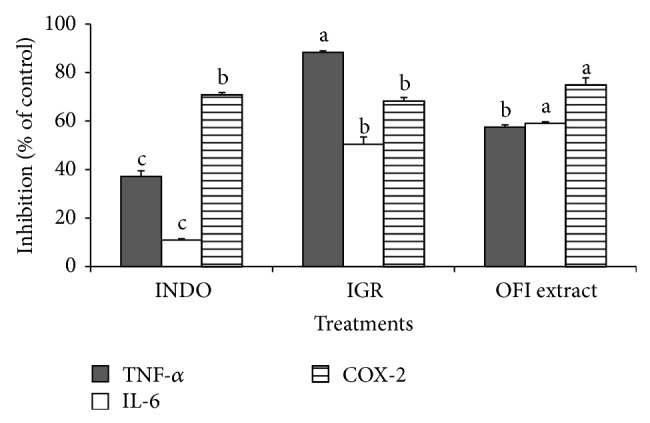
Effect of indomethacin (INDO), isorhamnetin-glucosyl-rhamnoside (IGR), and* Opuntia ficus-indica *(OFI) extract on TNF-*α* and interleukin- (IL-) 6 levels and on COX-2 activity in rat ears tissue homogenate. Indomethacin was used as positive control. Control group only received croton oil 5% v/v as irritant agent. Data represent means ± standard deviations of at least 6 replicates. ^a, b, c^Different letters denote significant differences among treatments (*P* < 0.05).

Proinflammatory cytokines TNF-*α* and IL-6 were determined in order to clarify the topical anti-inflammatory mechanisms of isorhamnetin glycosides present and isolated from the OFI extract (Figure 3). The greatest suppression of TNF-*α* production was observed in ears treated with IGR (88.3 ± 0.5%) while the OFI extract showed greater IL-6 suppression (59.1 ± 1.4%). The pure IGR increased TNF-*α* inhibition in 30.9% in contrast to the inhibition caused by the OFI crude extract. The OFI extract showed more effect as an inhibitor of COX-2 (74.8 ± 2.9%) than indomethacin and IGR which exerted the same inhibitory effect (70.9 ± 0.9% and 68.3 ± 0.6%, resp.).

## 4. Discussion 

The observed effects on cell viability were similar to those reported by Benayad et al. [23] who tested the anti-inflammatory activities of extracts from Moroccan* Opuntia ficus-indica* flowers. Cell viability of macrophage RAW 264.7 cells was ≥90% for all samples at the concentrations tested [23]. Therefore, reduction of NO was not due to the cytotoxicity of isorhamnetin glycosides since cell viability was above 80%.

All isolated isorhamnetin glycosides showed inhibitory effects higher than 50% on NO production even at concentrations as low as 25 ng IsoEq/mL. Isorhamnetin diglycosides had a higher anti-inflammatory potential than triglycosides. Other reports had demonstrated that kaempferol glycosides inhibited differently the NO production. Particularly, kaempferol 7-*O*-*α*-L-rhamnopyranoside showed an IC50 value of 41.2 *μ*M, whereas kaempferol triglycosides and diglycosides at the dosage of 100 *μ*M had an inhibitory effect of less than 10% [30]. Fang et al. [22] showed that kaempferitrin and kaempferol-3-*O*-*β*-D-apiofuranosyl-(1-2)-*α*-L-arabinofuranosyl-7-*O*-*α*-L-rhamnopyranoside differently inhibited the NO production exhibiting an IC50 of 40 *μ*M and 15 *μ*M, respectively.

Additionally, Rho et al. [31] demonstrated that the position and the number of sugar moieties affect the suppression of NO production exerted by flavonol glycosides. Kaempferol 7-*O*-*α*-L-rhamnopyranoside showed more potential to inhibit NO production (IC50 = 37.7 *μ*M) than kaempferol 3-*O*-*α*-L-rhamnopyranoside (IC50 > 100 *μ*M). Likewise, Rho et al. [31] showed that the presence of an additional rhamnose moiety at the 3-position caused a loss in NO inhibitory activity as in the case of kaempferol-3, 7-*O*-*α*-dirhamnoside (IC50 > 100 *μ*M). These results are consistent with our findings where the diglycoside IGR showed greater inhibitory activity on NO production compared with its corresponding triglycoside IGRR that has an additional rhamnose moiety.

Isorhamnetin glycosides had a higher inhibition on nitric oxide production compared to aglycone tested at 25 ng IsoEq/mL (Table 3). In contrast, previous reports indicated that a quercetin glycoside had a similar effect to the aglycone only when tested at a concentration 30 times higher [32]. But baicalein and baicalein-6-*α*-glucoside when they were tested at 40 *μ*M showed similar inhibitory activities on the NO production (approximately 60%) [33].

The effect of OFI extract at 50 ng IsoEq/mL was comparable with the NO production inhibition induced by an extract of* O. ficus-indica* flowers (0.5 mg extract/mL) in RAW 264.7 cell line (>60%) [23]. Isolated isorhamnetin glycosides showed greater NO production inhibition than OFI extract when tested at 25 ng IsoEq/mL. But, when concentration was increased to 125 ng IsoEq/mL, OFI extract inhibited the NO production similarly or more potently than the isorhamnetin glycosides tested. Kazłowska et al. [34] reported that* Porphyra dentata* crude extract was more active inhibiting NO production compared to its pure compounds (catechol, rutin, and hesperidin). Harasstani et al. [35] have demonstrated that when flavonoids are combined, as occurs in plant extracts, these can significantly increase their inhibitory effect upon NO production.

In the ear edema inflammation model, IGR and indomethacin showed the better effects. The effect of IGR was comparable with the 81% of inhibition induced by the tiliroside (flavonol acyl-glucoside) on an acute TPA-induced ear edema inflammation model [36]. As observed in the* in vitro* experiments, triglycoside IGRR showed lower anti-inflammatory effects compared with diglycosides and indomethacin. The difference in hydrophobicity between isorhamnetin diglycosides and triglycosides alters the cell-membrane permeability, which may be the reason for the better effect exerted by diglycosides. It has been suggested that the compounds with a MW < 500 Da are suitably transported across skin [37, 38].

Different anti-inflammatory effects were observed among isorhamnetin diglycosides, IGR and IGP, although they only differed in the pentose methylation. These results indicated that the type of sugar affected bioactivity, contrary to those reported by Sosa et al. [39] who showed that quercetin-3-*O*-galactoside and quercetin-3-*O*-glucoside at 1.0 *μ*moL/ear exhibited similar inhibition in a mouse ear edema induced by croton oil (52% and 51%, resp.). Erdemoglu et al. [40] demonstrated that quercetin-3-*O*-galactoside and quercetin-3-*O*-rhamnoside, tested at 0.5 mg/ear, inhibited differently the edema induced in mice by TPA (32% and 44.7%, resp.), suggesting that the presence of rhamnose could increase the anti-inflammatory potential.

Although some reports indicated that flavonoid glycosylation reduced the anti-inflammatory effect, isorhamnetin (ISO) and IGP showed similar effects on the rat ear edema inflammation model. This trend had been previously reported for hesperetin and the hesperetin-7-rutinoside that had similar anti-inflammatory effects (44% and 45%, resp.) in mice model of ear swelling induced by xylene [41].

The inhibitory effect of the OFI extract on ear edema was comparable to that produced by the chloroform extract from* Senna villosa* leaves (57.9%) in the TPA-induced ear edema model and slightly greater (52.4%) than the extract of* Pistacia khinjuk* L. leaves, which also was rich in glycosylated flavonoids [42, 43]. Some isolated compounds can be more potent anti-inflammatory drugs than their source extract. Mo et al. [28] reported that the MeOH extracts of* Lithraea molleoides* (Vell.) Engl. (Anacardiaceae) inhibited the ear edema induced by TPA by 21%, while the methyl gallate, one of the main compounds in MeOH extract, at an equivalent concentration, produced 63% of ear edema inhibition. Likewise, an ethanolic extract of* Myagropsis myagroides *(90 *μ*g/ear) markedly inhibited the phorbol myristate acetate- (PMA-) induced ear edema with 66.8% inhibition (*P* < 0.05), one-third of 6,6′-bieckol activity which was tested at 30 *μ*g/ear [44]. In this investigation, although the OFI extract showed an adequate inhibitory effect on ear edema, it did not exceed the effects observed with isorhamnetin diglycosides IGR and IGP. Based on these results, the order of anti-inflammatory potential against the ear edema was IGR = INDO > IGP = ISO > OFI extract > IGRR.

Tumor necrosis factor (TNF) is a potent mediator of inflammation with a powerful proinflammatory capacity, especially within immune cells capable of producing a cascade of downstream cytokines and chemokines expressed by monocytes/macrophages [45]. Meanwhile, IL-6 is an important factor for the synthesis of acute phase proteins, whose serum level is increased in acute and chronic inflammatory diseases such as cancer [46, 47]. In skin inflammation, cytokines such as TNF-*α* and IL-6 from resident cells keratinocytes bind to receptors on the vascular endothelium, activating cellular signaling pathways and inducing expression of vascular endothelial cell adhesion molecules [48].

Both IGR and OFI extract inhibited COX-2 activity and inflammatory cytokines IL-6 and TNF-*α*. TNF-*α* activation stimulates the IL-6 synthesis in several cell types and, in turn, IL-6 inhibits the TNF-*α* production providing a negative feedback, thus limiting the acute inflammatory response [49]. IGR showed more pronounced effect on TNF-*α* than IL-6 or COX-2 activity, indicating that its anti-inflammatory effect was mediated by the inhibition of proinflammatory cytokines. Differences in the cytokine modulation indicate that at least a part of the IL-6 production could be directed and not mediated by TNF-*α* activation [50]. Mueller et al. [51] observed that an apple extract containing 5% quercetin and 30% phloridzin (glycosylated phenolic compound) suppressed more potently the TNF-*α* production (89%) than that of IL-6 (32%) in a LPS-stimulated macrophage model.

The OFI extract showed greater inhibition on COX-2 activity than on TNF-*α* or IL-6. Inhibitory effects of OFI extract on TNF-*α* and IL-6 were similar to those observed for quercetin (59.1 ± 3.3% and 57.9 ± 9.9%, resp.) measured in human blood [52].

On the other hand, Lee et al. [53] proved that a flavonol-rich hexane fraction from* Rhus verniciflua* stokes and its major compound fisetin affected differently the TNF-*α* and IL-6 production* in vitro*. The* R. verniciflua* hexane fraction (1 *μ*g/mL), which contained about 0.0827 *μ*g/mL of fisetin, suppressed approximately 26.3% of the TNF-*α* production in ear edema. When pure fisetin was tested at 0.1 *μ*g/mL, the TNF-*α* inhibition increased. Likewise, a crude hydroalcoholic extract of* Croton antisyphiliticus* Mart. and its isolated glycosylated flavonoid (apigenin-*β*-*D*-glucopyranosyl) showed similar suppressing effect on TNF-*α* production despite the differences among the concentrations tested (100 mg/Kg and 10 mg/Kg, resp.) [54]. Results presented herein agree with previous reports for glycosylated flavonoids such as the tiliroside, wogonoside, kaempferol-3-*O*-glucoside, quercetin 3-*O*-glucoside, and isorhamnetin-3-*O*-glucoside as important inhibitors of TNF-*α* and IL-6 production [55–58].

Anti-inflammatory effects of flavonoids can occur through different molecular mechanisms such as inhibition of histamine release from basophils, inhibition of proinflammatory eicosanoids production, or by inhibiting the production of proinflammatory cytokines [23]. In this study it was demonstrated that isorhamnetin glycosides and the OFI extract modulate the inflammatory response by suppressing the COX-2 activity and also by the reduction in levels of NO, IL-6, and TNF-*α* both* in vitro* and* in vivo*.

## 5. Conclusions

The* Opuntia ficus-indica* (OFI) extract and its isorhamnetin glycosides exerted anti-inflammatory effects.* In vitro*, isorhamnetin diglycoside IGR and the OFI extract significantly reduced the nitric oxide production in RAW 264.7 macrophage cells without significantly affecting viability, while,* in vivo*, IGR showed an efficacy comparable to indomethacin to reduce rat ear edema induced by croton oil.

The attached sugar moiety affected the* in vivo* anti-inflammatory effects of the diglycosides IGP and IGR. Likewise, the glycosylation degree affected the bioactivity, the triglycoside IGRR being less bioactive compared to the diglycoside IGR. The topical anti-inflammatory effect of IGR was mediated by the suppression of COX-2 activity and inhibition on the production of proinflammatory cytokines such as TNF-*α* and IL-6.

Further research is needed in order to determine the permeability and absorption of these compounds and evaluate their efficacy in dermatological preparations. Additional experiments are required to validate the mechanism of action, including iNOS expression and other genes involved in the inflammatory response.

## Supplementary Material

Along with the molecular ion, sodium adducts were obseved in each mass spectra. Ionization conditions allowed the detection of fragments generated by the loss of three, two and one sugar moieties observed in the triglycosides mass spectrum (Figure S1); likewise, fragments generated by the loss of two and one sugar moieties were observed in the diglycosides mass spectrum (Figure S2). The m/z [317.05] corresponding to isorhamnetin aglycone appears in all mass spectrum.

## Figures and Tables

**Table 1:** Identification and quantification of most abundant isorhamnetin glycosides associated with *Opuntia ficus-indica *extract*. *

ID compound	Tentative structure	Concentration (*µ*moL/g)^*^	UV/VIS *λ*max (nm)	[M+H] *m*/*z *	References
IGRR	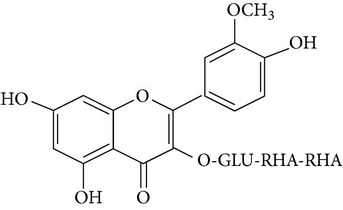	227.6 ± 7.5	253, 353	771	[12–14]
IGRP	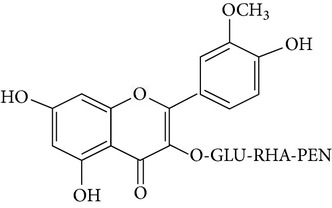	220.2 ± 6.4	253, 354	757	[12–14]
IGP	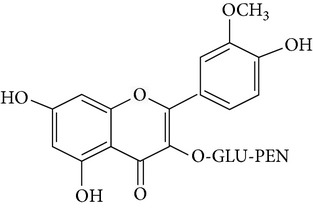	93.1 ± 3.3	253, 353	611	[12–14]
IGR	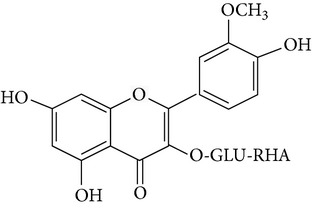	95.1 ± 5.9	254, 354	625	[12–14]

^*^Concentration expressed in *µ*moL of IsoEq/g of OFI extract. IGRR: isorhamnetin-glucosyl-rhamnosyl-rhamnoside; IGRP: isorhamnetin-glucosyl-rhamnosyl-pentoside; IGP: isorhamnetin-glucosyl-pentoside; IGR: isorhamnetin-glucosyl-rhamnoside.

**Table 2:** Effect of *Opuntia ficus-indica* (OFI) extract, isorhamnetin, and its glycosides in RAW 264.7 cell viability.

Treatment	Cell viability (%)		
	25 ng/mL^#^	50 ng/mL	125 ng/mL
Isorhamnetin	99.3 ± 5.6^a∗^	97. 5 ± 5.3^a^	97.7 ± 2.1^a^
IGP	96.7 ± 2.4^ab^	88.9 ± 5.3^b^	84.9 ± 4.0^cd^
IGR	92.2 ± 3.1^b^	85.2 ± 5.1^b^	81.6 ± 3.0^d^
IGRR	99.4 ± 2.9^a^	98.5 ± 2.3^a^	95.7 ± 4.5^ab^
IGRP	99.4 ± 3.9^a^	97.5 ± 2.8^a^	94.2 ± 4.0^ab^
OFI extract	94.5 ± 1.2^ab^	92.6 ± 4.8^ab^	89.9 ± 1.9^bc^

^#^Concentration expressed in ng IsoEq/mL. IGP: isorhamnetin-glucosyl-pentoside; IGR: isorhamnetin-glucosyl-rhamnoside; IGRR: isorhamnetin-glucosyl-rhamnosyl-rhamnoside; IGRP: isorhamnetin-glucosyl-rhamnosyl-pentoside. ^*^Different letters in each column denote significant differences among treatments (*P* < 0.05).

**Table 3:** Percentage of inhibition of nitric oxide production in RAW 264.7 cells incubated with different concentrations of *Opuntia ficus-indica *(OFI) extract, isorhamnetin, and its glycosides.

Treatment	Nitric oxide inhibition (%)		
	Concentration (ng IsoEq/mL)		
	25	50	125
Isorhamnetin	19.6 ± 2.2^Cd^	47.6 ± 5.8^Bc^	55.5 ± 3.1^Ac^
IGP	57.4 ± 9.1^Aab^	58.7 ± 4.2^Aab^	60.7 ± 4.3^Abc^
IGR	60.0 ± 3.4^Ba^	65.4 ± 4.2^ABa^	68.7 ± 5.0^Aab^
IGRR	50.6 ± 4.0^Bb^	54.2 ± 2.8^Bbc^	61.2 ± 5.8^Abc^
IGRP	53.2 ± 4.7^Aab^	54.4 ± 6.3^Abc^	59.9 ± 4.1^Ac^
OFI extract	40.8 ± 4.5^Cc^	57.2 ± 5.7^Bab^	73.5 ± 4.8^Aa^

IGP: isorhamnetin-glucosyl-pentoside; IGR: isorhamnetin-glucosyl-rhamnoside; IGRR: isorhamnetin-glucosyl-rhamnosyl-rhamnoside; IGRP: isorhamnetin-glucosyl-rhamnosyl-pentoside.

^a,b,c^Different lower case letters denote significant differences among treatments.

^A,B,C^Different upper case letters denote significant differences among concentrations (*P* < 0.05).

## Abbreviations

IsoEq: Isorhamnetin equivalents
OFI: * Opuntia ficus-indica*.
