# Supplementary material for: Topical Anti-Inflammatory Effects of Isorhamnetin Glycosides Isolated from Opuntia ficus-indica
Source: Biomed Res Int. 2015 Mar 2;2015:847320. doi: 10.1155/2015/847320 (PMC4363586; doi:10.1155/2015/847320)
Supplement: Supplementary file 1 — Along with the molecular ion, sodium adducts were obseved in each mass spectra. Ionization conditions allowed the detection of fragments generated by the loss of three, two and one sugar moieties observed in the triglycosides mass spectrum (Figure S1); likewise, fragments generated by the loss of two and one sugar moieties were observed in the diglycosides mass spectrum (Figure S2). The m/z [317.05] corresponding to isorhamnetin aglycone appears in all mass spectrum. [file 847320.f1.pdf]

A

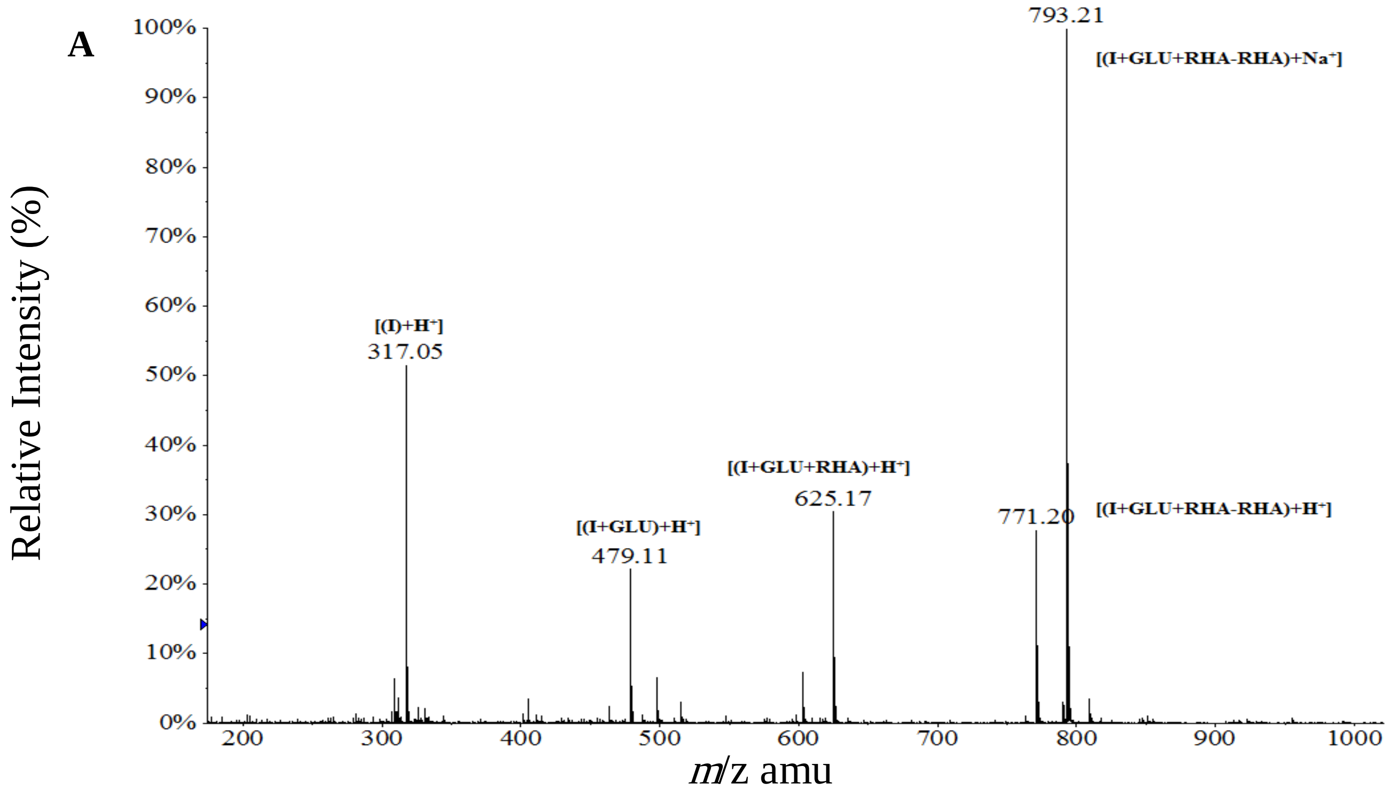

B

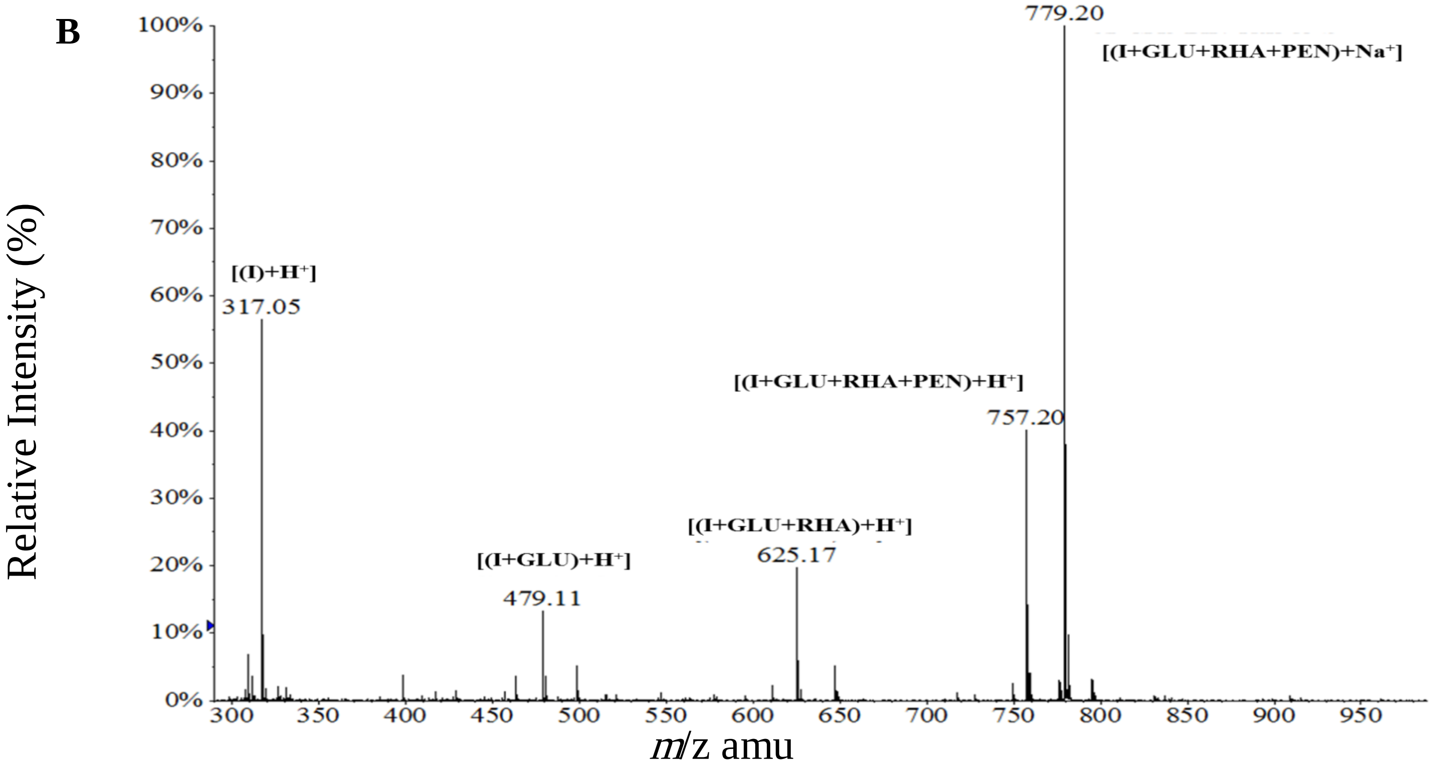

Fig. S1. Fragmentation pattern of IGRR (A) and IGRP (B) by LC/MSD TOF analysis (ESI+). I: Isorhamnetin; GLU: Glucose; RHA: Rhamnose; P: Pentose.

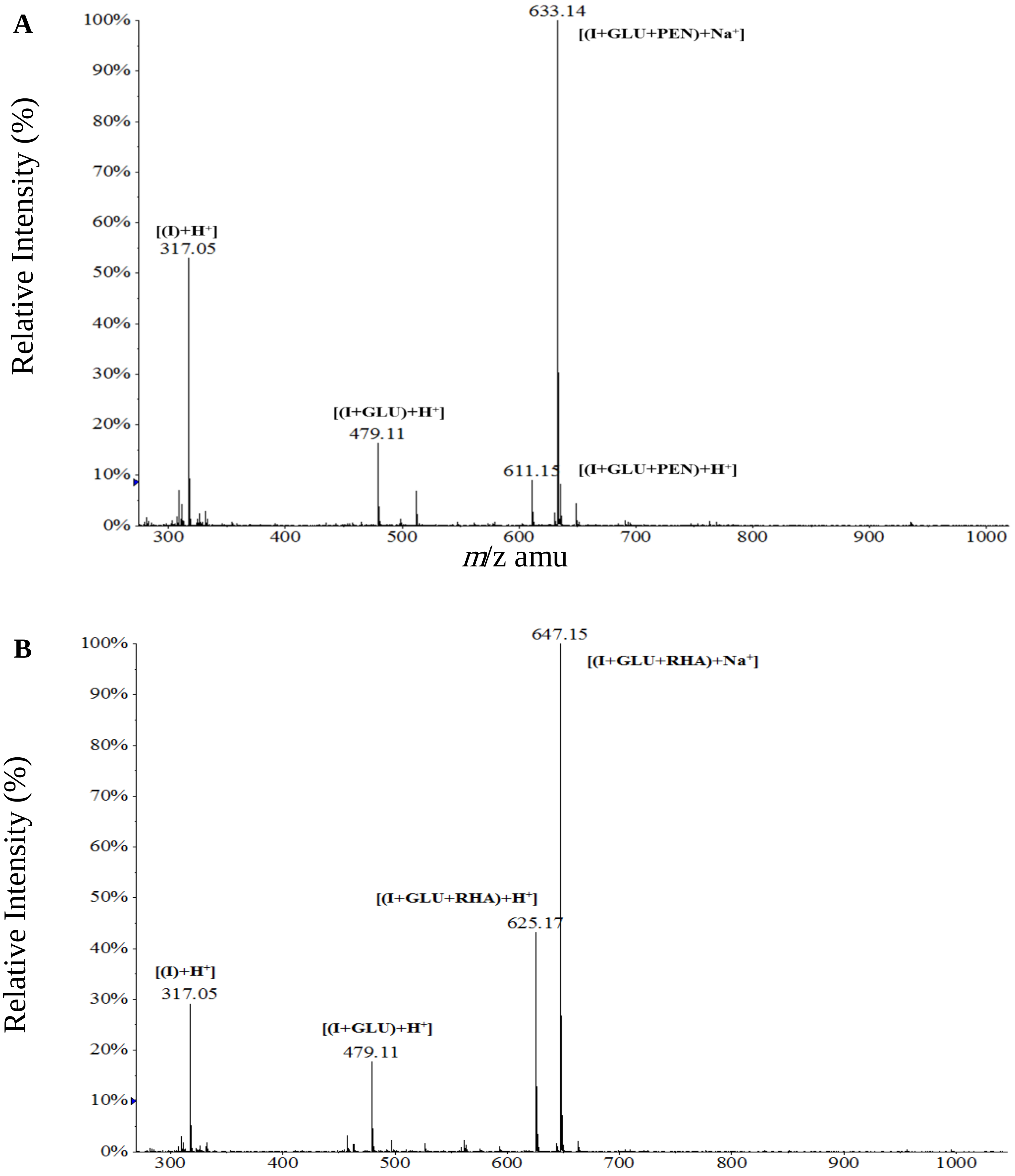

Fig. S2. Fragmentation pattern of IGP (A) and IGR (B) by LC/MSD TOF analysis (ESI+). I: Isorhamnetin; GLU: Glucose; RHA: Rhamnose; P: Pentose.
